# Neuro-Physiotherapy Regimen to Enhance the Functional Performance of a Hemiplegic Patient Following Brain Tumor Resection: A Case Report

**DOI:** 10.7759/cureus.30421

**Published:** 2022-10-18

**Authors:** Nikita Kaple, Pallavi Harjpal, Snehal S Samal

**Affiliations:** 1 Physiotherapy, Ravi Nair Physiotherapy College, Datta Meghe Institute of Medical Sciences, Wardha, IND; 2 Kinesiology, Ravi Nair Physiotherapy College, Datta Meghe Institute of Medical Sciences, Wardha, IND

**Keywords:** physiotherapy, tumor, who grade iii glioma, hemiplegia, anaplastic astrocytoma

## Abstract

Anaplastic astrocytoma is a kind of astrocytoma (a type of brain cancer) that is classified as World Health Organization (WHO) grade III. Headaches, poor mental status, focal neurological impairments, and seizures are the most prevalent early signs and symptoms of anaplastic astrocytoma. Anaplastic astrocytomas have also been linked to earlier exposure to vinyl chloride and large doses of brain radiation treatment. Anaplastic astrocytomas are a form of astrocytoma that also falls under the umbrella term of gliomas, which are tumors that develop from glial cells. This is because astrocytes are a kind of glial cell. As a result, anaplastic astrocytomas (grade III) are also known as "grade III gliomas" or "high-grade gliomas. In this case study, we present a case of a 35-year-old male who presented to our cancer hospital with complaints of weakness in the right upper and lower limbs for two years. He was then diagnosed with anaplastic astrocytoma, grade III. But after tumor resection, he developed right hemiplegia with involvement of the right upper extremity and lower extremity. This case study demonstrates how the neuro-physiotherapy rehabilitation protocol in the case of hemiplegia after brain tumor resection aids in improving motor function and functional independence. Physiotherapy treatment that is modulated according to the patient’s needs plays a vital role in improving the quality of life and helping to delay the worsening of symptoms, thereby helping to increase the life span of patients diagnosed with anaplastic astrocytoma grade III.

## Introduction

Anaplastic astrocytoma (AA) is a malignant astrocytic primary brain tumor that is diffusely invading. It is an uncommon disease in the adult population that constitutes 4% of all malignant central nervous system (CNS) tumors and 10% of all gliomas. Astrocytomas are tumors that grow from astrocytes, which are star-shaped brain cells [1]. Glioblastoma multiforme and anaplastic astrocytoma are expected to affect five to eight people per 100,000 in the general population. Anaplastic astrocytomas are most common in people between the ages of 30 and 50 [2]. Astrocytomas are classified into four groups by the World Health Organization (WHO) based on their rate of development and proclivity for spreading (infiltrating) to adjacent brain tissue. Low-grade astrocytomas are benign and are classified as grade I or II astrocytomas. Grade III and grade IV astrocytomas are malignant and are also known as high-grade astrocytomas. Anaplastic astrocytomas are grade III astrocytomas [3]. A grade III astrocytoma is also called an anaplastic (malignant) astrocytoma because it grows faster than a grade II astrocytoma. Anaplastic astrocytoma frequently exhibits symptoms like headaches, lethargy, sleepiness, vomiting, and changes in personality or mental state [4].

Anaplastic astrocytomas can develop in any portion of the central nervous system, but they're most prevalent in the cerebrum, the large spherical section of the brain that occupies the majority of the skull [5]. According to researchers [6, 7], environmental factors (e.g., UV rays, certain chemicals, ionizing radiation), diet, stress, and/or other factors may all have a role in the development of certain cancers [6,7].

## Case presentation

Patient information

We present the case of a 35-year-old male with right-handed dominance. He presented with complaints of weakness in the right upper and lower limbs, which he had developed two months prior. He had had a history of tobacco use since 2003. The patient then came to our hospital on January 21, 2022, as he started experiencing right upper and lower limb weakness. He was provided with conservative treatment. Then he visited again on March 7, 2022, for his presenting complaints and was instructed to undertake specific tests, such as an MRI, which was completed on March 8, 2022. During those investigations, it was determined that he had an anaplastic astrocytoma grade III, for which he underwent excision. Postoperatively, he developed right hemiparesis. He was referred to the physiotherapy division after being admitted to our cancer center 1.5 months prior.

Clinical findings

The patient was assessed in a supine position. During the examination, a higher mental function was expected (the score was 26/30 on the Mini-mental Scale Examination). Cranial nerves VIII, V, VII, and VIII were impaired. On sensory analysis, the superficial, deep, and cortical sensations were intact bilaterally over the upper and lower limbs along with the trunk. His deep tendon reflexes (DTR) are demonstrated in Table 1. The planter was an extensor on the right side. The tone assessment done during the motor examination is shown in Table 2.

**Table 1:** Grading of deep tendon reflexes.

Deep tendon reflexes	Right	Left
Bicep jerk	1+(Diminished)	2+(Normal)
Triceps jerk	1+	2+
Knee jerk	1+	2+
Ankle jerk	1+	2+

**Table 2:** Tone assessment.

Tone	Right	Left
Upper limbs	Increased	Normal
Lower limbs	Reduced	Normal

Physiotherapy interventions

The patient received regular physiotherapy for six weeks, once a day. The Brunnstrom stage of recovery is shown in Table 3.

**Table 3:** Outcome measures

	Day 1	Week 4	Week 8
Modified Ashworth scale	Flaccid	1+	Normal tone
Vectorcardiography	0 LL 1 UL	2	5
Brunnstrom’s recovery stages	1 LL 1 UL	2	5

**Figure 1:**
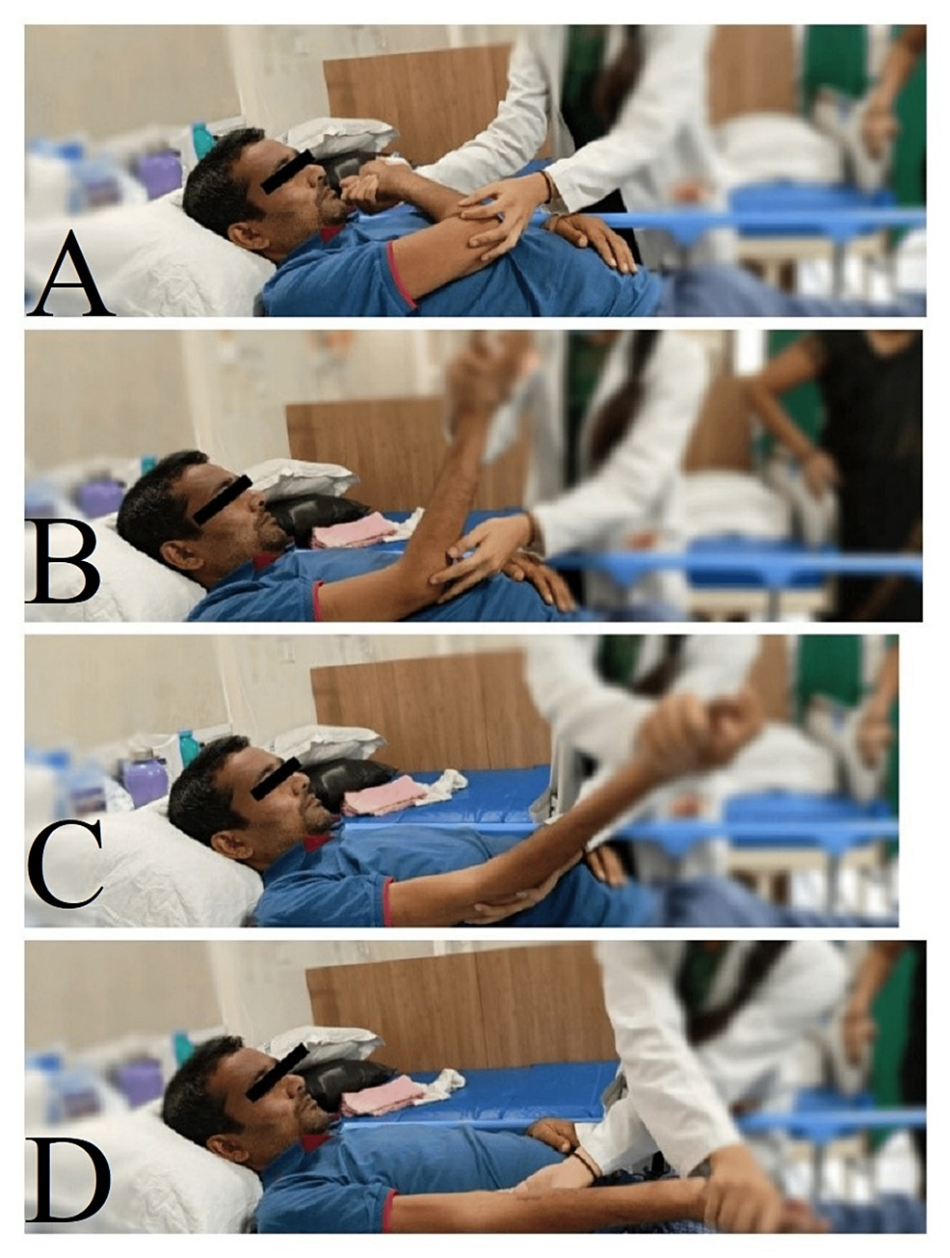
The therapist helping the patient perform the upper limb PNF pattern passively. A: PNF pattern D1 (initial position) with shoulder adduction and external rotation B: PNF pattern D1 (forearm supination) C: PNF pattern D1 (wrist flexion) D: PNF pattern D1 (fingers flexion)

**Figure 2:**
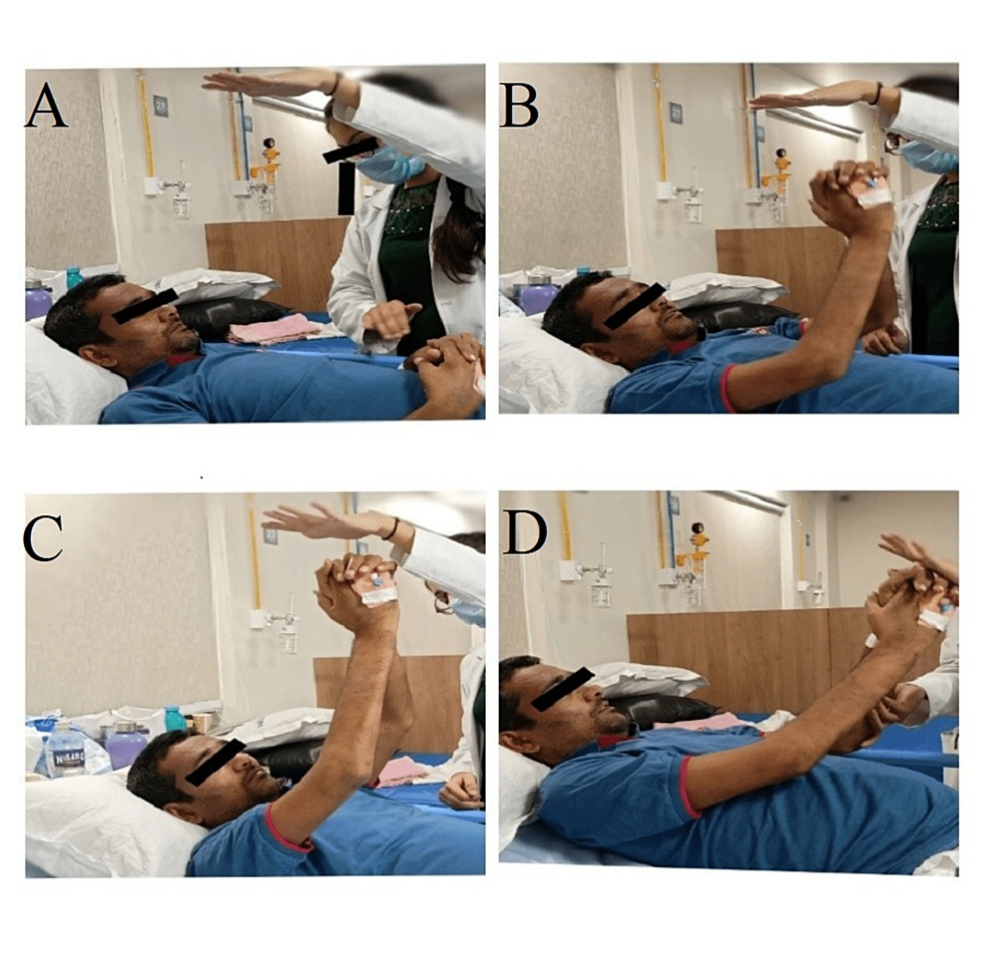
The patient is seen performing the reach-out exercise actively. A: Providing a target to the patient B: Patient initiating movement C: Patient trying to reach the target D: Therapist helping the patient maintain the reach

He was also taught simple exercises to be performed in the evening. The detailed intervention is provided in Table 4, Figure 1, and Figure 2. 

**Table 4:** Physiotherapy protocol representing the intervention, rationale, and strategy. UL: upper limbs, LL: lower limbs

Intervention	Rationale	Strategy
Supine-to-sit transition training with rolling facilitation	To improve bed mobility and functional status. To prevent secondary complications such as pressure sores and contractures.	Rolling facilitation: rolling from supine to side-lying using upper extremity momentum and crossing the ankles. Transition training from supine to sitting: Prone on the elbows from prone on elbows to long sitting, use elbow walking. Supine on elbows.
Proprioceptive Neuromuscular Facilitation (PNF)	To improve the range and strength of UL and LL muscles. To improve coordination in functional activities.	PNF for UL (Figure 2) and LL, flexion-adduction-external rotation, D1 extension-abduction-internal rotation, flexion-abduction-external rotation, D2 extension-adduction-internal rotation.
Bedside sitting	Assist in improving sitting balance and training sit-to-stand transfer.	The patient sits with her feet supported, her hips and knees flexed to 90 degrees, her trunk neutral, and her elbows locked. The weight shift in sitting and reach-outs in supine (Figure 3) and sitting were also added.
Sit-to-stand transfer	Strengthens the muscles of LL.	Sit to stand with holds.
Standing	Activities performed while standing help to improve standing balance.	Standing with a parallel bar as support, spot marching, and weight shifting.
Gait training	Helps to normalize the gait pattern.	Walking with verbal feedback, stepping up and down, as well as walking around obstacles.
Facial exercises	Strengthen the facial muscles.	Raising eyebrows, smiling, blowing a candle, puffing cheeks, and clenching teeth (actively assisted).

**Figure 3:**
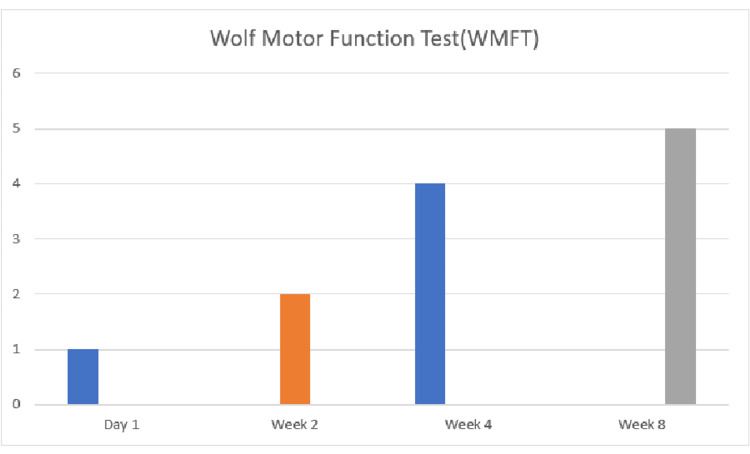
Progression in Wolf Motor Function Test score.

**Figure 4:**
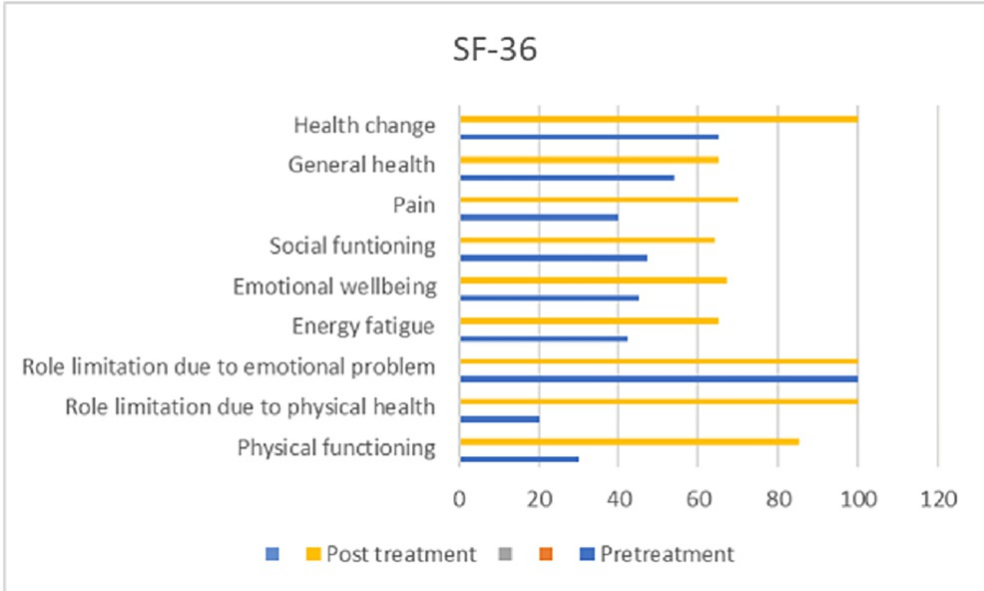
Progression in SF-36 which measures health-related quality of life.

There was a commendable improvement in the patient post-rehabilitation. There was normalization in muscle tone (graded by the Modified Ashworth Scale) and recovery in the Brunnstrom stage of recovery. There was also an improvement in the Wolf motor function test (WMFT) (Figure 3) and SF-36 (Figure 4). The WMFT has 17 elements in the most generally used form. The first six items are timed functional activities; the seventh and fourteenth items are strength tests, and the final nine items entail assessing movement quality while performing various tasks.

## Discussion

A brain tumor has been demonstrated to be a risk factor for acute ischemic infarction and vice versa. Ischemic stroke patients are more likely to develop brain tumors, usually gliomas, as a result of alterations in the cell's functional and metabolic status caused by ischemia and hypoxia [8]. On the other hand, tumor-related metastasis, a general prothrombotic tendency, and proliferating cell mass increase the risk of ischemic infarcts. Potential mechanisms implicated in the interaction between the two processes include astrocyte activation, reactive gliosis, angiogenesis, and other alterations in the tumor microenvironment, all of which are predominantly induced by cerebral ischemia as a result of glioma development. Moreover, the frequent resection procedures used to treat gliomas raise the risk of ischemic damage [9].

Many cancer patients suffer from physical dysfunction as a result of chemotherapy, radiation therapy, and surgery, with impairments in muscle strength, flexibility, and endurance. Physical therapy is a comprehensive multidisciplinary approach to the identification and management of astrocytoma patients [10]. Physical therapy can help with issues including weakness, tense soft tissues, stiff joints, weariness, and edema, among others. Physical therapy-assisted exercise has been shown to enhance cancer patients' quality of life [11]. Physical therapy has been shown to help with tiredness, muscular strength, and exercise capacity. Significant functional improvement was seen in BT patients who received comprehensive rehabilitation for motor, balance, cognition, and activities of daily living (ADL) function. This was comparable to the improvement seen in stroke patients [12]. Aerobic and resistance exercises are recommended to improve muscle strength and endurance. Resistance training of intact muscles is recommended to compensate for impaired coordination [13]. Many studies have shown a significant improvement in core stability. As a result of this, gait and daily activities changed in patients who used the PNF technique during their rehabilitation therapy, both on the affected side and the healthy side [14].

Consequently, patients need long-term integrated and coordinated management, including rehabilitation, to improve their functional, mental, and emotional well-being and quality of life. According to reports, people with BT can achieve functional improvements comparable to those with stroke and TBI (traumatic brain injury) when undergoing inpatient rehabilitation. Physiotherapy and occupational therapy are recommended by the Australian Cancer Network (ACN) for patients with residual motor deficits (strength, coordination, and balance) and residual issues in self-care and functional independence, respectively. To improve muscle strength and endurance, aerobic and resistance exercises for all BT patients are suggested. To compensate for impaired coordination, it encourages resistance training of intact muscles [15].

## Conclusions

Following brain tumor removal, motor dysfunction is likely to develop and may cause functional dependence, decreased mobility, difficulties doing everyday tasks, a higher chance of developing mobility-related issues, falls, pain, anxiety, or depression, as well as poor quality of life.
